# Transcriptome profiling analysis of uterus during chicken laying periods

**DOI:** 10.1186/s12864-023-09521-z

**Published:** 2023-08-03

**Authors:** Tiantian Sun, Cong Xiao, Zhuliang Yang, Jixian Deng, Xiurong Yang

**Affiliations:** 1 College of Animal Science and Technology, Guangxi University, Nanning, 530004 China; 2 Guangxi Key Laboratory of Animal Breeding, Disease Control and Prevention, Guangxi University, Nanning, 530004 China

**Keywords:** Uterus, Chicken, RNA-Seq, Eggshell, Laying period

## Abstract

The avian eggshell is formed in the uterus. Changes in uterine function may have a significant effect on eggshell quality. To identify the vital genes impacting uterine functional maintenance in the chicken, uteri in three different periods (22W, 31W, 51W) were selected for RNA sequencing and bioinformatics analysis. In our study, 520, 706 and 736 differentially expressed genes (DEGs) were respectively detected in the W31 vs W22 group, W51 vs W31 group and W51 vs W22 group. Gene Ontology (GO) and Kyoto Encyclopedia of Genes and Genomes (KEGG) analysis indicated DEGs were enriched in the extracellular matrix, extracellular region part, extracellular region, extracellular matrix structural constituent, ECM receptor interaction, collagen-containing extracellular matrix and collagen trimer in the uterus (*P* < 0.05). Protein–protein interaction analysis revealed that *FN1*, *LOX*, *THBS2*, *COL1A1*, *COL1A2*, *COL5A1*, *COL5A2*, *POSTN*, *MMP13*, *VANGL2*, *RAD54B*, *SPP1*, *SDC1*, *BTC*, *ANGPTL3* might be key candidate genes for uterine functional maintenance in chicken. This study discovered dominant genes and pathways which enhanced our knowledge of chicken uterine functional maintenance.

**Supplementary Information:**

The online version contains supplementary material available at 10.1186/s12864-023-09521-z.

## Introduction

In the uterus, the egg absorbs fluid into albumin, which then calcifies on the eggshell membrane to form the eggshell. The formation of eggshells is a highly complex process that is precisely controlled by genetic and biological pathways in the uterus [1–3]. Eggshell formation requires the involvement of multiple ion transporter genes to supply the required ions and minerals [4]. ATP binding cassette subfamily C member 9 (*ABCC9*), inositol 1,4,5-trisphosphate receptor type 2 (*ITPR2)*, potassium inwardly rectifying channel subfamily J member 8 (*KCNJ8)* and WNK lysine deficient protein kinase 1 (*WNK1)* modulate eggshell thickness by participating in ion transport [5]. Eggshells contain a variety of matrix proteins [6–8]. Three matrix protein genes ovocleidin-11, ovalbumin and ovocalyxin-32 were shown to affect eggshell thickness [9]. Milk fat globule EGF and factor V/VIII domain containing (*MFGE8*) and EGF like repeats and discoidin domains 3 (*EDIL3*) are two glycoproteins involved in the regulation of vesicle-mediated eggshell calcification [10]. These proteins and other organic components interact with minerals to form the eggshell in the uterus [11–13].

Eggshell quality decreases as hens age in the laying period [14–17]. Aged hens have a disrupted uterine structure with reduced glandular density, fibrosis and atrophy of the endometrium, probably due to continued egg-laying behavior, which leads to reduced protein synthesis, ion transport and immune defense of the uterus [17, 18]. The expression of matrix proteins and ion transporters (BPI fold containing family B member 3, ATPase sarcoplasmic/endoplasmic reticulum Ca^2+^ transporting 2, ovalbumin, carbonic anhydrase 2, transferrin, sodium channel epithelial 1 gamma subunit, ovocleidin 17) in the uterus of laying hens change with age [19–21]. Age-related dysregulation of gene expression in uterus may lead to deterioration of eggshell quality.

The rapid development of RNA sequencing (RNA-seq) has provided a powerful tool for the study of the transcriptome [22, 23]. RNA-seq has been widely used in the study of various species and traits in recent years [24–29]. There have been many studies on poultry uterus over the years [3, 30–32]. However, the avian uterine functional differences in different laying periods at the transcriptome level remains obscure. In this study, we screened differentially expressed genes and pathways in chicken uterus at different laying stages by RNA-seq and bioinformatics analysis, which laid a foundation for further studies on uterine function maintenance in chicken.

## Materials and methods

### Animals and sample collection

Twelve Nandan-Yao hens (*Gallus gallus*) used in this study, laying continuously 3 eggs, were sourced from ﻿Guangxi Guigang Gangfeng Agriculture and Husbandry Co., Ltd. Euthanasia was performed by cervical dislocation, with all effort made to minimize suffering. All chickens were caged and reared individually according to standard feeding management protocols. The uterine samples were obtained from three periods ﻿(four chickens per group), including early laying period (22 weeks old), peak laying period (31 weeks old), and late laying period (51 weeks old).

### Total RNA extraction

The total RNA was extracted from the uterus with TRIzol reagent (﻿Invitrogen Life Technologies, USA) ﻿following the manufacturer's instruction. Agarose gel electrophoresis was used to assess RNA concentration. RNA integrity was determined using UV–Vis Spectrophotometer Q5000 (Quawell, USA).

### RNA sequencing and quality control

The cDNA libraries were constructed and sequenced following the manufacturer’s standard procedures on an Illumina HiSeq 2500 (Illumina, San Diego, CA, USA) in Novogene Bioinformatics Technology Co., Ltd., Beijing, China. Raw reads of FASTQ format were processed with the Trim Galore [33]. Low quality sequences including rate of N base > 10%, quality score < 20 and adaptor sequences were removed to generate clean data for downstream analysis. FastQC software was used to calculate sequence duplication levels, GC content and Q20 scores of clean data [34].

### Bioinformatics analysis

Reference genome and gene model annotation files were downloaded from the ENSEMBL (http://ftp.ensembl.org/pub/release-102/gtf/gallus_gallus/, http://ftp.ensembl.org/pub/release-102/fasta/gallus_gallus/dna/). The clean reads were.mapped to the chicken reference genome by Hisat2v2.1.0 [35, 36]. The stringtiev2.1.1 was then used to annotate the transcripts [37]. ﻿Differential expression analysis was performed with the DESeq2 [38], a package in R software. Differentially expressed genes (DEGs) were screened by *P*-value < 0.05 and |log_2_foldchange|≥ 1. Gene Ontology (GO) and Kyoto Encyclopedia of Genes and Genomes (KEGG) [39–41] analysis were conducted by the R package clusterProfiler 3.14.3 to identify critical pathways [42]. *P*-adjust < 0.05 denoted statistical significance. Interactions between DEGs were explored by STRING [42] database, with confidence score > 0.9 being valid [43]. We applied Cytoscape [44] (http://cytoscape.org/) to visualize the STRING analysis results and CytoHubba application to find key hub genes through the MCC algorithm [45]. Module detection (MCODE) in Cytoscape was used to screen the key modules in the protein–protein interaction network [46].

### Validation of RNA-Seq

﻿Six genes retinoic acid receptor responder 1 (*RARRES1*), dickkopf WNT signaling pathway inhibitor 3(*DKK3*), R-spondin 3(*RSPO3*), relaxin 3(*RLN3*), glypican 4(*GPC4*) and potassium inwardly rectifying channel subfamily J member 2(*KCNJ2*) related to uterine functional maintenance were selected for qRT-PCR analysis to validate RNA-seq data [9, 47–50]. Primer sequences of target and reference genes were shown in Supplemental table 1. RNA was reverse transcribed into cDNA using RT Reagent Kit (Takara, Dalian, China). ﻿qRT-PCR was performed by SYBR Green Supermix kit (Takara, Dalian, China) in Bio-RAD CFX96 Real Time Detection system. The qRT-PCR was performed according to the following program: the volume of the reaction mixture was 20 μl, with 2 μl of cDNA, 0.4 μl of primers, 10 μl of SYBR (Takara, Dalian, China), and 7.2 μl of RNA-free water. Then the QRT-PCR was run at 95 ^◦^C for 30 s, 95 ^◦^C for 5 s, 60 ^◦^C for 30 s, 35 cycles. The expression levels of genes were calculated relative to the expression of the *β-actin* using the 2^−ΔΔCT^ method.

## Results

### Transcriptome data

As shown in Supplementary table 2, a total of 270,440,113 clean reads were acquired, ranging from 19,122,152 to 26,346,160. The clean reads were characterized by the average GC content of 51.95% and more than 97.12% of Q20, while more than 90.59% of clean reads were mapped to the reference genome.﻿

### Analysis of differentially expressed genes

**Fig. 1:**
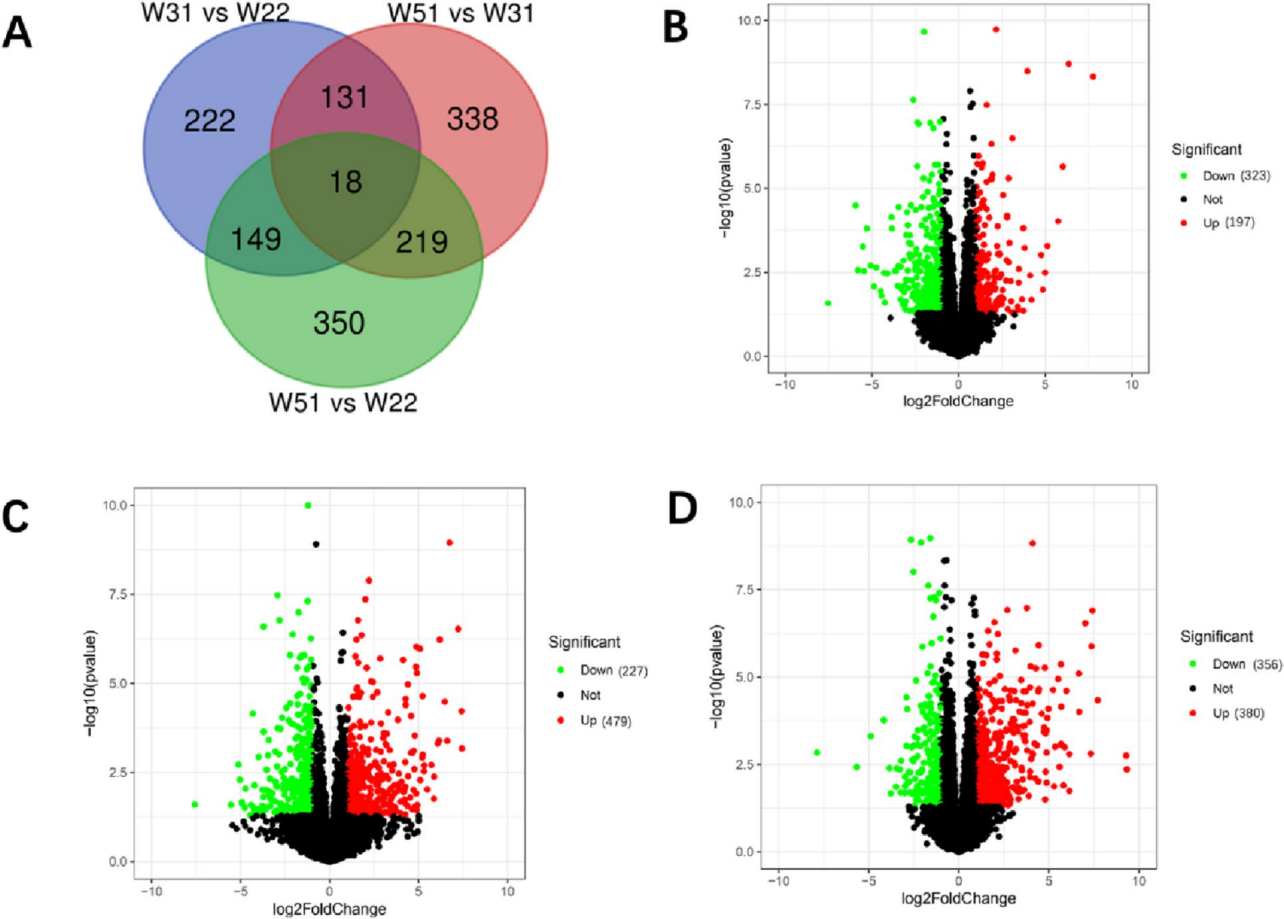
Venn diagram and Volcano plot of DEGs. **A** represents the Venn diagram of intersection DEGs of W31 vs W22, W51 vs W31 and W51 vs W22. **B**, **C** and **D** represent the volcano plot of DEGs in W31 vs W22, W51 vs W31 and W51 vs W22, respectively

There were 520, 706 and 736 DEGs identified by comparison of the uterus in W31 vs W22 group, W51 vs W31 group, W51 vs W22 group, respectively (Fig. 1A). Of the DEGs, 197 up-regulated and 323 down-regulated genes in the W31 vs W22 group (Fig. 1B), 479 up-regulated and 227 down-regulated genes in the W51 vs W31(Fig. 1C), 380 up-regulated and 356 down-regulated genes in the W51 vs W22(Fig. 1D). Furthermore, the intersection of the three comparison groups showed a total of 18 DEGs including sodium voltage-gated channel beta subunit 3 (*SCN3B*), avidin (*AVD*), family with sequence similarity 178*,* member B (*FAM178B*), solute carrier family 17 member 9 (*SLC17A9*), unc-13 homolog C (*UNC13C*), multiple EGF-like-domains 6-like(*LOC424998*), proopiomelanocortin (*POMC*), serum/glucocorticoid regulated kinase 1 *(SGK1)*, ADAM metallopeptidase with thrombospondin type 1 motif 18*(ADAMTS18)*, HEAT repeat-containing protein 8-like (*HRC8L*), transglutaminase 4 (*TGM4*), transmembrane protein 2-like *(LOC415478)*, amphiregulin (*AREG*), ligand of Numb protein X 2-like (*LOC422320*), protein kinase cGMP-dependent type II (*PRKG2*) and so on (Fig. 1A).

### GO and KEGG analysis for DEGs

**Fig. 2:**
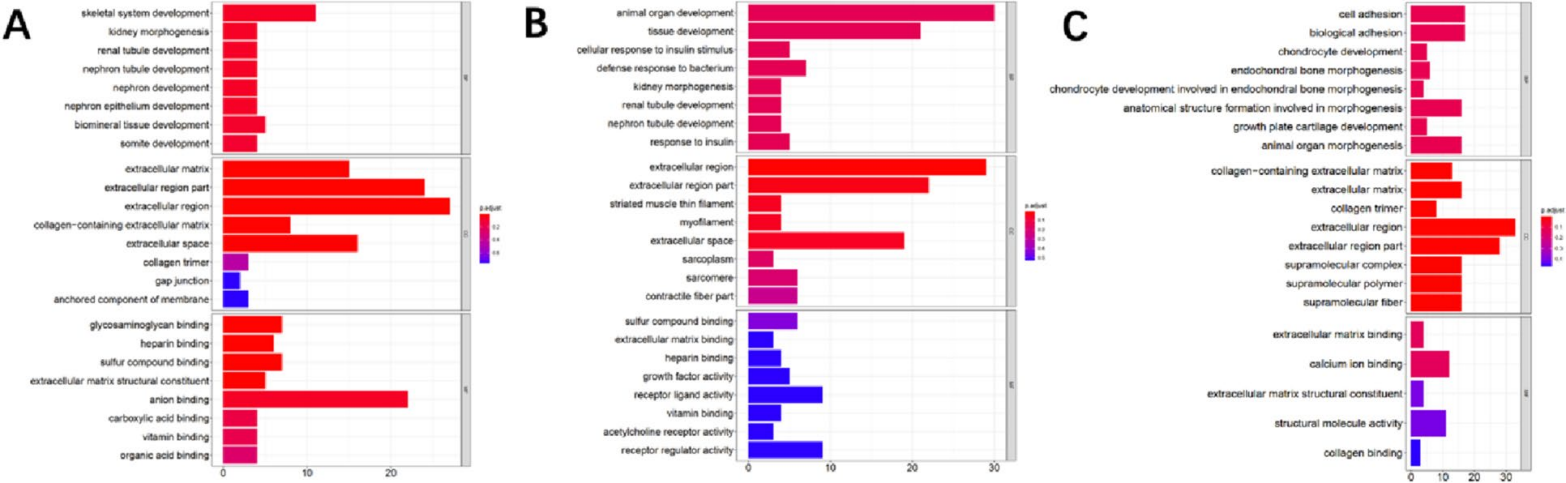
GO enrichment analysis of DEGs. **A**, **B** and **C** represent GO enrichment analysis of DEGs in W31 vs W22, W51 vs W31 and W51 vs W22, respectively

In the W31 vs W22 group, GO analysis indicated that DEGs were enriched in 9 terms, including extracellular matrix, extracellular region part, extracellular region, collagen-containing extracellular matrix, extracellular space, glycosaminoglycan binding, heparin binding, sulfur compound binding and extracellular matrix structural constituent (Fig. 2A, Supplementary table 3).

**Fig. 3:**
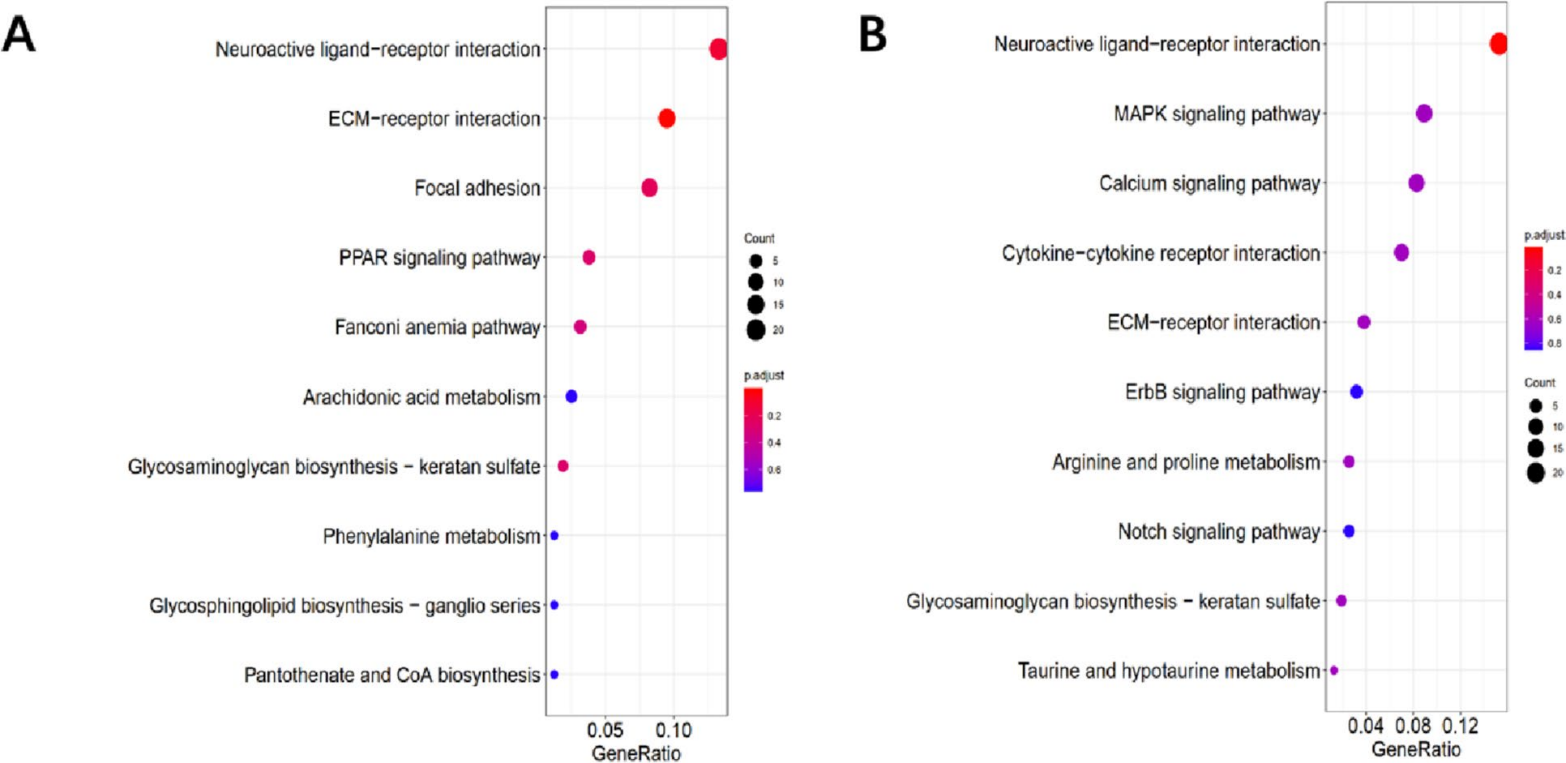
KEGG enrichment analysis of DEGs. **A** and **B** represent KEGG analysis of DEGs in W51 vs W31, W51 vs W22, respectively

The results of functional enrichment analysis of the W51 vs W31 group showed that the extracellular region and extracellular region part were significantly enriched in GO analysis (Fig. 2B, Supplementary table 3), and neuroactive ligand-receptor interaction were significantly enriched in KEGG analysis (Fig. 3A, Supplementary table 5).

In the W51 vs W22 group, GO analysis of DEGs were significantly enriched in 9 terms, containing collagen-containing extracellular matrix, extracellular matrix, collagen trimer, extracellular region, extracellular region part, supramolecular complex, supramolecular polymer, supramolecular fiber and extracellular space (Fig. 2C, Supplementary table 6). And ECM-receptor interaction was significantly enriched in KEGG analysis (Fig. 3B, Supplementary table 7).

### Integration of PPI network

**Fig. 4:**
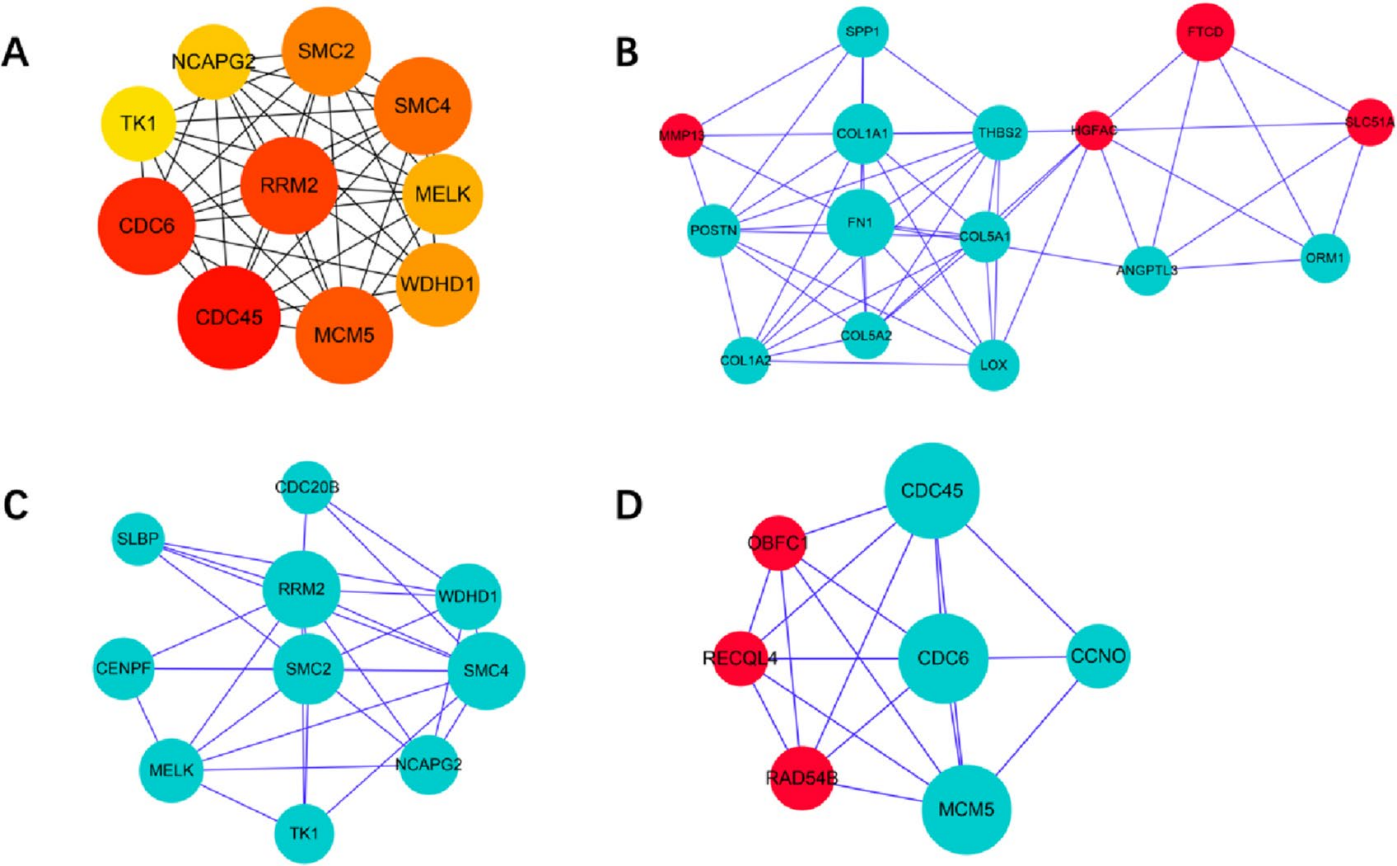
Top ten genes and three protein–protein interaction (PPI) hub network modules of W31 vs W22 group. **A** represents the top ten genes of W31 vs W22. The darker the color of the node represents the higher ranking of the gene. **B**, **C** and **D** represent the top three modules, respectively. Red and blue nodes indicate up- and down-regulation of genes, respectively. Larger nodes represent more interaction relationships

In the W31 vs W22 group, the top ten genes were cell division cycle 45(*CDC45*), cell division cycle 6(*CDC6*), ribonucleotide reductase regulatory subunit M2(*RRM2*)*,* minichromosome maintenance complex component 5(*MCM5*), structural maintenance of chromosomes 4(*SMC4*), structural maintenance of chromosomes 2 (*SMC2*), WD repeat and HMG-box DNA binding protein 1(*WDHD1*), maternal embryonic leucine zipper kinase(*MELK*), non-SMC condensin II complex subunit G2(*NCAPG2*), and thymidine kinase 1(*TK1*) (Fig. 4A). Module analysis showed modules 1, 2 and 3 included 15, 10 and 7 genes with module scores of 11.091, 6.333 and 6.25, respectively(Fig. 4B-D).

**Fig. 5:**
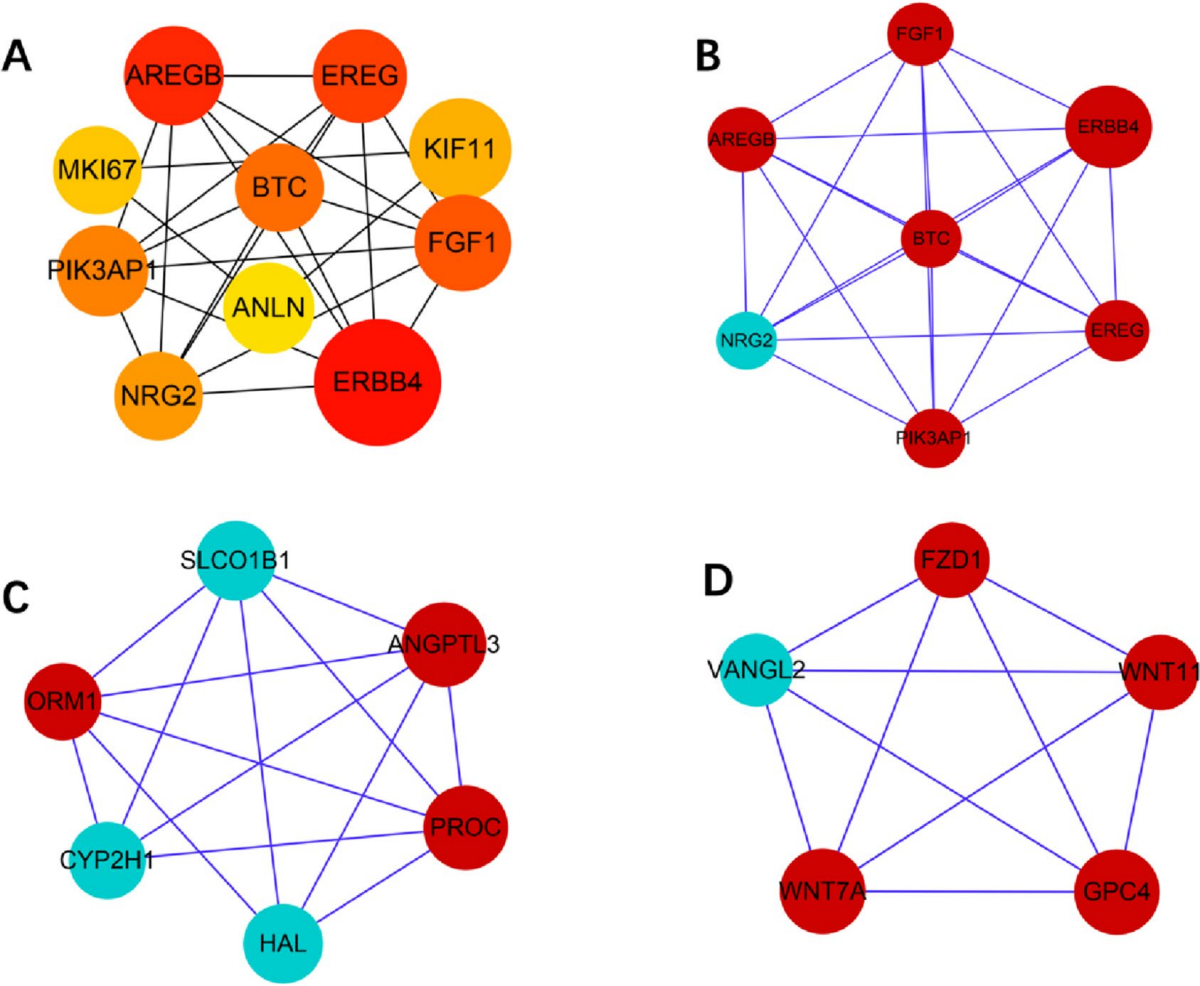
Top ten genes and three protein–protein interaction (PPI) hub network modules of W51 vs W31 group. **A** represents the top ten genes of W51 vs W31. The darker the color of the node represents the higher ranking of the gene. **B**, **C** and **D** represent the top three modules, respectively. Red and blue nodes indicate up- and down-regulation of genes, respectively. Larger nodes represent more interaction relationships

The top ten genes were erb-b2 receptor tyrosine kinase 4(*ERBB4*), amphiregulin (*AREGB*), epiregulin (*EREG*), fibroblast growth factor 1(*FGF1*), betacellulin (*BTC*), phosphoinositide-3-kinase adaptor protein 1(*PIK3AP1*), neuregulin 2(*NRG2*), kinesin family member 11(*KIF11*), marker of proliferation Ki-67(*MKI67*) and anillin actin binding protein (*ANLN*) in the W51 vs W31 group (Fig. 5A). Module analysis indicated modules 1, 2 and 3 contained 7, 6 and 5 genes with module scores of 7.143, 6.444 and 6, respectively (Fig. 5B-D).

**Fig. 6:**
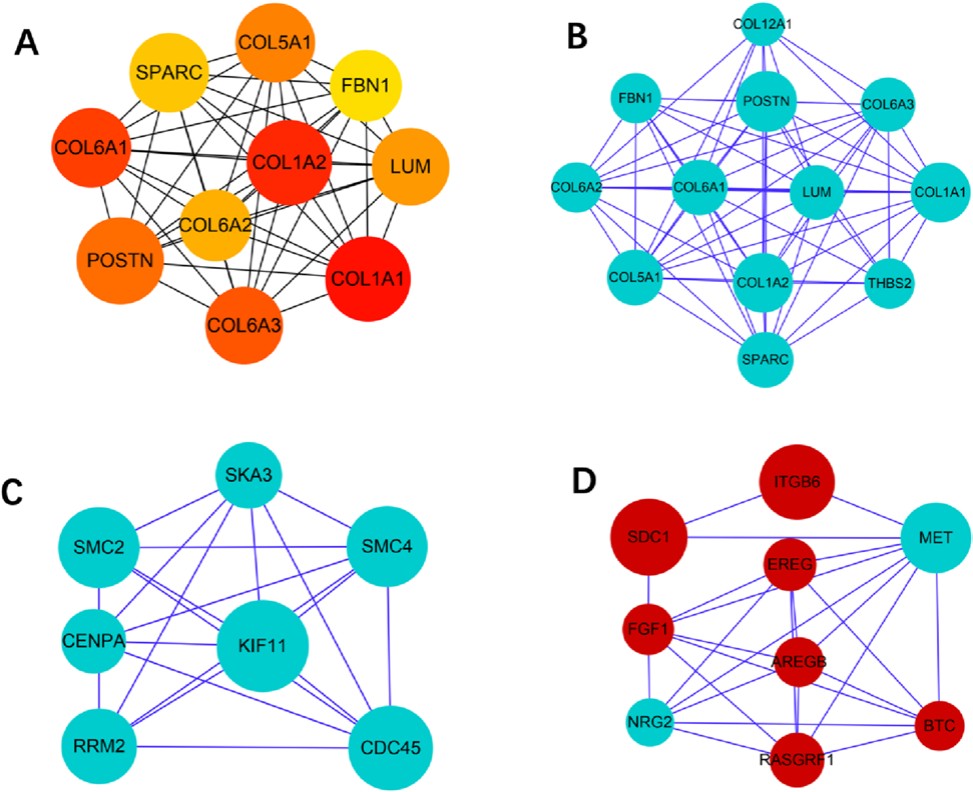
Top ten genes and three protein–protein interaction (PPI) hub network modules of W51 vs W22 group. **A** represents the top ten genes of W51 vs W22. The darker the color of the node represents the higher ranking of the gene. **B**, **C** and **D** represent the top three modules, respectively. Red and blue nodes indicate up- and down-regulation of genes, respectively. Larger nodes represent more interaction relationships

In the W51 vs W22 group, the top ten genes contained collagen type I alpha 1 chain(*COL1A1*), collagen type I alpha 2 chain(*COL1A2*), collagen type VI alpha 1 chain(*COL6A1*), collagen type VI alpha 3 chain(*COL6A3*), periostin *(POSTN)*, collagen type V alpha 1 chain(*COL5A1*), lumican (*LUM*), collagen type VI alpha 2 chain(*COL6A2*), secreted protein acidic and cysteine rich (*SPARC*), and fibrillin 1(*FBN1*) (Fig. 6A). Module analysis revealed modules 1, 2 and 3 consisted of 12, 7 and 9 genes with module scores of 6, 4 and 3, respectively (Fig. 6B-D).

### Dynamic transcriptional profiling of the uterus

**Fig. 7:**
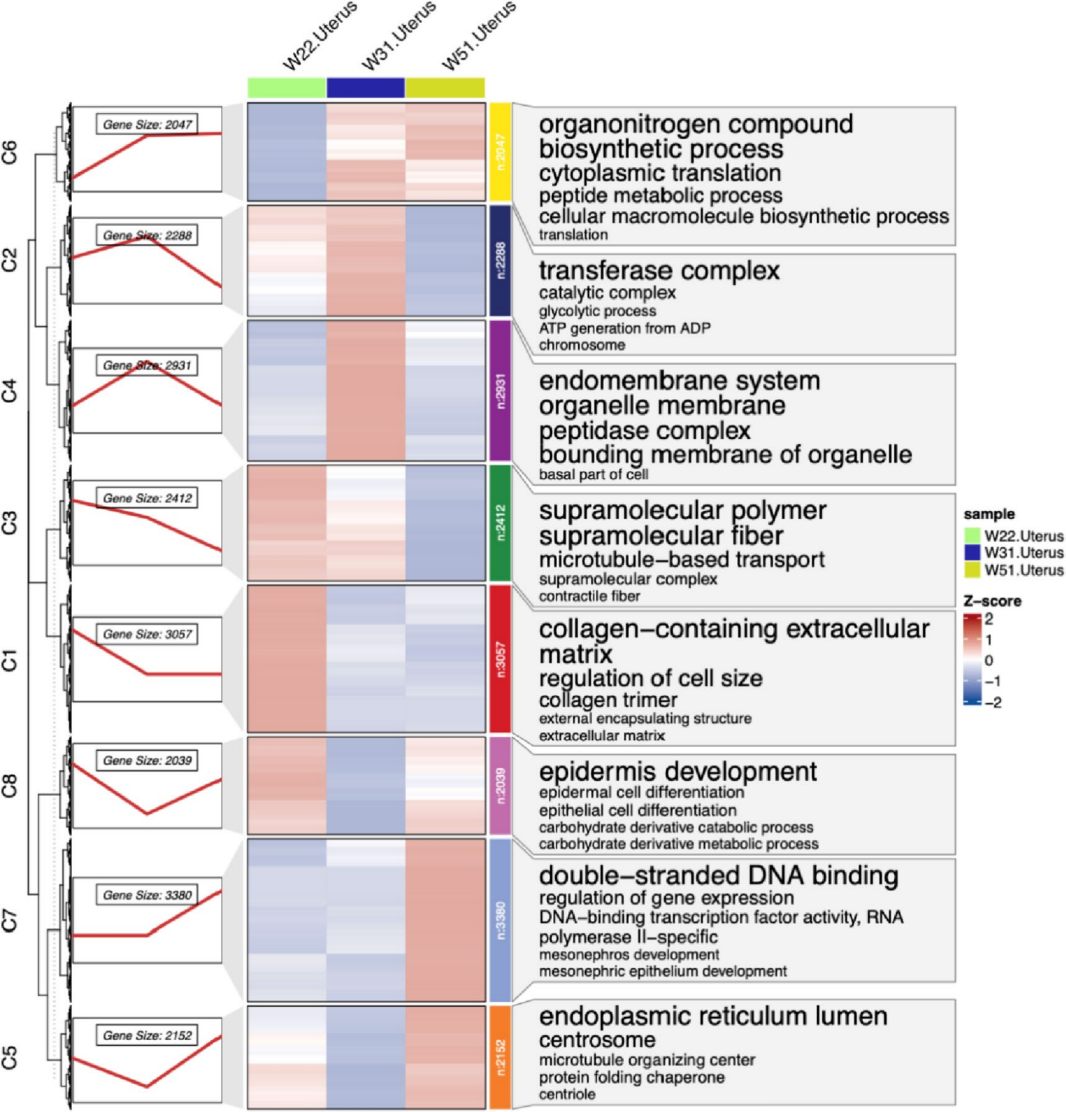
Dynamic transcriptional profiling of the uterus. Note: Left: Time series expression spectrum of eight clusters. The red line indicates the average gene expression level. Middle plot: Expression heatmap plotted with TPM values for each gene in eight clusters. Right: Top five significant gene GO enrichment in each cluster

To study the dynamic transcriptome profile of chicken uterus during the laying period (22W, 31W, 51W), eight major expression patterns were determined using the fuzzy c-means algorithm in the Mfuzz package, and the results are shown in Fig. 7.

Cluster 1 (3057 genes), cluster 3 (2412 genes) and cluster 8 (2039 genes) were specifically highly expressed at 22W. Cluster 1 significantly enriched in 147 GO terms. which mainly includes collagen-containing extracellular matrix, regulation of cell size, collagen trimer, and external encapsulating structure, extracellular matrix, etc. Cluster 3 significantly enriched in 34 GO terms, which mainly includes supramolecular polymer, supramolecular fiber, microtubule-based transport, supramolecular complex, contractile fiber, etc. Cluster 8 significantly enriched in 76 GO terms, which mainly includes epidermis development, epidermal cell differentiation, epithelial cell differentiation and carbohydrate derivative catabolic process, carbohydrate derivative metabolic process, etc. KEGG enrichment analysis showed that these genes were significantly enriched in cytokine-cytokine receptor interaction signaling pathway.

Cluster 2 (2288 genes) and cluster 4 (2931 genes) were specifically highly expressed at 31W. Cluster 2 significantly enriched in 89 GO terms, which mainly includes transferase complex, catalytic complex, glycolytic process, ATP generation from ADP, chromosome, etc. KEGG enrichment analysis showed that these genes were significantly enriched in the proteasome signaling pathway. Cluster 4 significantly enriched in 14 GO terms, which mainly includes endomembrane system, organelle membrane, peptidase complex, bounding membrane of organelle, basal part of cell and so on. KEGG enrichment analysis showed that these genes in cluster 4 were significantly enriched in 2 signaling pathways, including oxidative phosphorylation and proteasome. Cluster 6 (2047 genes) was highly expressed at 31W and 51W. KEGG enrichment analysis showed that these genes were significantly enriched in the ribosome signaling pathway.

Cluster 5 (2152 genes) and cluster 7 (3380 genes) were specifically highly expressed at 51W. Cluster 5 significantly enriched in 9 GO terms, which mainly includes endoplasmic reticulum lumen, centrosome, microtubule organizing center, protein folding chaperone, centriole, etc. Cluster 7 significantly enriched in 175 GO terms, which mainly includes double-stranded DNA binding, regulation of gene expression, DNA-binding transcription factor activity, RNA polymerase II-specific, mesonephros development, mesonephric epithelium development, etc. KEGG enrichment analysis showed that these genes in cluster 7 were significantly enriched in 2 signaling pathways, including MAPK signaling pathway and neuroactive ligand-receptor interaction.

### Validation of RNA-seq

**Fig. 8:**
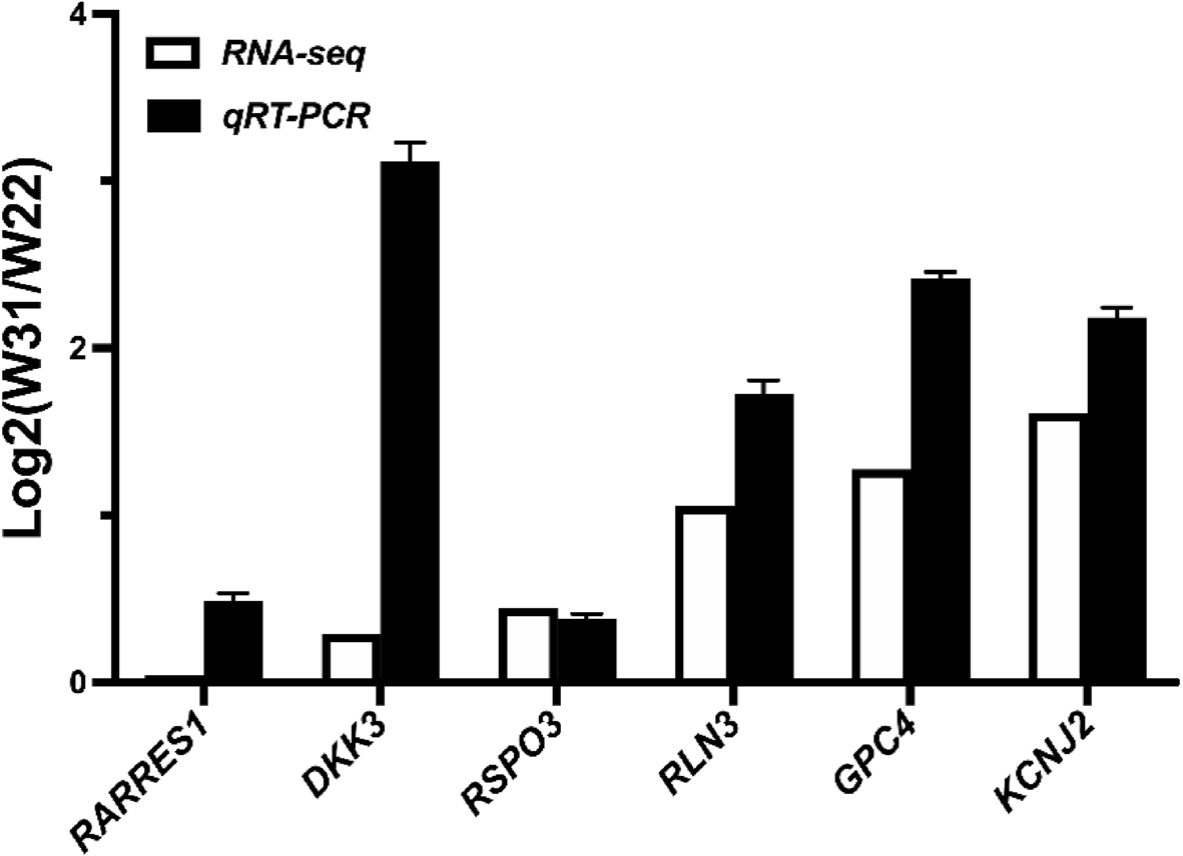
RNA-Seq validation using QRT-PCR. Six DEGs (*RARRES1*, *DKK3*, *RSPO3*, *RLN3*, *GPC4*, *KCNJ2*) were used to validate the accuracy of RNA sequencing. *N* = 4

We chose 6 genes (*RARRES1*, *DKK3*, *RSPO3*, *RLN3*, *GPC4*, *KCNJ2*) related to uterine functional maintenance for qRT-PCR analysis to validate RNA-seq data. The analysis data showed that the trends of six genes were corroborated with RNA-seq analysis results(Fig. 8). The results indicated that the results of RNA-seq analysis were accurate and reliable.

## Discussion

The structure and function of the uterus have a critical impact on eggshell quality. Ion transport and eggshell matrix protein secretion in the uterus are regulated by numerous genes [2, 51, 52]. In this study, high-throughput transcriptome analysis was used to investigate the differential gene expression profiles in three different periods of uterus. The identified differentially expressed genes and pathways generated by this study are a rich resource that can be used to deepen our understanding of functional maintenance of the chicken uterus.

DEGs were significantly enriched in the extracellular matrix, extracellular region, extracellular region part, ECM receptor interaction, extracellular matrix structural constituent, collagen-containing extracellular matrix and collagen trimer. The extracellular matrix (ECM) is involved in uterine function [53]. The ECM consists of collagen, proteoglycans and glycoproteins such as fibronectin and laminin, which is a complex mixture of structural proteins that act as a support structure to maintain three-dimensional tissue architecture. The ECM also regulates cellular responses through ligand-integrin interactions, participating in cell adhesion, survival, proliferation, differentiation and migration [54–56].

Protein network interaction analysis of DEGs identified 15 genes related to uterine functional maintenance including collagen type V alpha 2 chain(*COL5A2*), *COL5A1, COL1A1, COL1A2*, fibronectin 1(*FN1*), lysyl oxidase (*LOX*), thrombospondin 2(*THBS2*), periostin (*POSTN*), matrix metallopeptidase 13(*MMP13*), VANGL planar cell polarity protein 2(*VANGL2*), RAD54 homolog B (*RAD54B*), secreted phosphoprotein 1(*SPP1*), Syndecan-1 (*SDC1*), Betacellulin (*BTC*), and angiopoietin like 3(*ANGPTL3*).

In our results, *FN1*, *LOX*, *THBS2*, *COL1A1*, *COL1A2*, *COL5A1*, *COL5A2* and *POSTN* were specifically highly expressed at 22W.Studies have shown that collagens are major structural protein components of the extracellular matrix. Collagen type V (COLV) acts in conjunction with collagen type I (*COL1A1*, *COL1A2*) and collagen type III to regulate fiber formation and deposition in the ECM [57]. There are three different isomers of COLV: *COL5A1*, *COL5A2* and *COL5A3 *[58]. *FN1* is a glycoprotein in the extracellular matrix in plasma and cell surface. It mediates cellular interactions with the ECM and takes part in cell adhesion, migration, growth, differentiation and metastasis [59, 60]. *FN1* plays an important role in the endometrium, influencing placentation, blastocyst adhesion and implantation [61–63]. *LOX* is a copper-containing amine oxidase that catalyzes cross-linking of collagen and elastin in the ECM. The inhibition of *LOX* activity decreases endometrial stromal cell migration and embryo adhesion [64]. *THBS2* is a homologous glycoprotein that is linked to the ECM disulfide bond. It is involved in adhesion, migration and proliferation of cell, interactions of cell-to-cell and cell–matrix, and angiogenesis [65, 66]. Our results showed that *COL1A1*, *COL1A2*, *THBS2*, *LOX*, *FN1* were significantly enriched in terms such as extracellular matrix, collagen-containing extracellular matrix, external encapsulating structure, extracellular matrix, extracellular region, extracellular space, etc. *POSTN* is a matricellular protein that can combine with structural components of the extracellular matrix which is abundantly expressed in collagen-rich fibrous connective tissue [67].

In our results, *MMP13*, *VANGL2* and *RAD54B* were specifically highly expressed at 31W. Matrix metallopeptidases (MMPs) and their inhibitors (TIMPs) regulate COL turnover and ECM remodeling [68]. Matrix metalloproteinases belong to the zinc proteases family that hydrolyze components of the extracellular matrix [69]. *MMP13* participates in the degradation of the collagen network of the ECM [69, 70]. *VANGL2* is the vertebrate ortholog of core Planar cell polarity (PCP) components. PCP refers to the capacity of a tissue, typically, but not exclusively, an epithelium, to transmit directional information across the tissue plane such that its cellular constituents can differentiate, divide or move in a coordinated manner and along a common axis, generally orthogonal to the apical-basal axis [71]. Research has shown that *VANGL2* gene play a crucial role in regulating planar cell polarity and convergent extension movements [72]. *RAD54B* involved in the initial stages of homologous recombination. Vertebrate cells lacking *RAD54B* exhibit reduced levels of gene conversion [73, 74]. *RAD54B* is involved in cell cycle regulation after DNA damage and participates in homologous recombinational repair, which ensures the precise repair of the most deleterious DNA lesions, double-stranded breaks [75]. The Peak laying period is accompanied by degradation of the collagen network of the ECM, homologous recombination, and cellular information transfer.

In our results, *SPP1*, *SDC1*, *BTC* and *ANGPTL3* were specifically highly expressed at 51W. *SPP1* is a secretory protein present in the extracellular matrix. *SPP1* protein is localized to the endometrium and participates in the formation and calcification of the eggshell [76–78]. Considering the reported inhibitory function of osteopontin in other mineralized systems, together with its main occurrence in the non-mineralized parts of the eggshell and at the outermost part of the shell, suggests that this molecule could be part of the mechanism regulating the eggshell calcification [79]. *SDC1* play an important role in the placenta, in trophoblast development, and in pregnancy complications, highlighting one of the most important members of this family. Proteoglycan macromolecules play key roles in several physiological processes (e.g., adhesion, proliferation, migration, invasion, angiogenesis, and apoptosis), all of which are important for placentation and healthy pregnancy [80]. *BTC* was shown to be expressed exclusively at the sites of blastocyst apposition in the mouse uterus, suggesting an involvement in embryo implantation [81]. Retarded (but not impaired) embryo implantation was observed in BTC-overexpressing transgenic mice [82]. *ANGPTL3* was an endometrial gene associated with angiogenesis, increasing uterine blood flow during early pregnancy to enhance the availability of micronutrients for transport into the uterine lumen [83]. These genes may interact to regulate the functional maintenance of the uterus.

## Conclusions

The results of the present study suggest show that uterine tissues of hens at different laying stages differ in their transcriptomes. A series of key genes and pathways involved in the maintenance of uterine function were identified using RNA-seq and bioinformatics analysis. These key genes (*FN1*, *LOX*, *THBS2*, *COL1A1*, *COL1A2*, *COL5A1*, *COL5A2*, *POSTN*, *MMP13*, *VANGL2*, *RAD54B*, *SPP1*, *SDC1*, *BTC*, *ANGPTL3*) may take part in uterine functional maintenance through the extracellular matrix, extracellular region, extracellular region part, ECM receptor interaction, extracellular matrix structural constituent, collagen-containing extracellular matrix and collagen trimer. We have generated a rich resource, which will provide insight into chicken uterine functional maintenance.

### Supplementary Information

**Additional file 1. **

**Additional file 2. **

**Additional file 3. **

## Data Availability

The RNA sequencing data used during the current study are available from the NCBI (accession number: PRJNA849016 and the link of the website: http://www.ncbi.nlm.nih.gov/bioproject/849016).

## Abbreviations

RNA-Seq: RNA sequencing
DEGs: Differentially expressed genes
GO: Gene Ontology
KEGG: Kyoto Encyclopedia of Genes and Genomes
qRT-PCR: Quantitative Real-time PCR
