# Supplementary material for: Transcriptome profiling analysis of uterus during chicken laying periods
Source: BMC Genomics. 2023 Aug 3;24:433. doi: 10.1186/s12864-023-09521-z (PMC10398974; doi:10.1186/s12864-023-09521-z)
Supplement: Supplementary file 1 — Additional file 1. [file 12864_2023_9521_MOESM1_ESM.docx]

**Supplementary table 1 qPCR Primer sequences**

| **Gene** | **Genbank accession no.** | **Primer sequence (5’-3’)** | **Melting temperature** | **Amplification length (bp)** |
| --- | --- | --- | --- | --- |
| RARRES1 | NM_204534.5 | F: GAAGGACAATGCTGTTGCGTT  R: GGTGACCTAATGTCCACACG | 59.0℃ | 104 |
| DKK3 | NM_205125.2 | F: CGAGATGTTGAATGCTGCGG  R: GGGTTGCAGTCATGTTGGTTC | 61.0℃ | 110 |
| RSPO3 | NM_001319025.3 | F: CGGCATGAAACAGATCGGAG  R:ACAATCAGCTTTACATTTTGCACAC | 61.0℃ | 103 |
| RLN3 | NM_001113200.2 | F: GAGTTCATCCGTGCCGTCAT  R: TGCTGCTTGAGAAGAATCGGC | 61.5℃ | 105 |
| GPC4 | XM_015278449.4 | F: GCCAAGAGCAGGTACGACT  R: TGCCGACGAATCACCATGTC | 58.5℃ | 110 |
| KCNJ2 | NM_205370.2 | F: ATCGGCTATGGCTTCAGGTG  R: GCGTCAATGATGCAGCCTAC | 59.5℃ | 92 |
| β-actin | NM_205518.1 | F: TATTGCTGCGCTCGTTGTTG  R: GGGCGACCCACGATAGATG | 60.8℃ | 102 |
