# Supplementary material for: Transcriptome profiling analysis of uterus during chicken laying periods
Source: BMC Genomics. 2023 Aug 3;24:433. doi: 10.1186/s12864-023-09521-z (PMC10398974; doi:10.1186/s12864-023-09521-z)
Supplement: Supplementary file 2 — Additional file 2. [file 12864_2023_9521_MOESM2_ESM.docx]

**Supplementary table 2. Summary of sequencing reads mapping to the reference genome and quality parameters**

| **Sample name** | **Raw reads** | **Clean reads** | **Q20(%)** | **GC content(%)** | | **Total mapped (%)** |
| --- | --- | --- | --- | --- | --- | --- |
| W22-U1 | 25,043,804 | 23,827,938 | 97.15 | | 51.97 | 90.95 |
| W22-U2 | 21,183,899 | 20,195,095 | 97.27 | | 52.07 | 90.59 |
| W22-U3 | 20,367,307 | 19,337,902 | 97.15 | | 51.81 | 91.06 |
| W22-U4 | 20,042,718 | 19,122,152 | 97.12 | | 51.58 | 92.37 |
| W31-U1 | 21,911,044 | 20,696,967 | 97.47 | | 51.96 | 91.80 |
| W31-U2 | 26,160,877 | 24,686,567 | 97.27 | | 51.92 | 91.38 |
| W31-U3 | 22,825,773 | 21,497,940 | 97.25 | | 52.48 | 90.89 |
| W31-U4 | 24,118,129 | 23,461,156 | 97.53 | | 52.16 | 90.82 |
| W51-U1 | 25,226,408 | 24,393,846 | 97.66 | | 51.78 | 90.86 |
| W51-U2 | 22,945,635 | 21,816,161 | 97.35 | | 51.99 | 91.80 |
| W51-U3 | 27,990,620 | 26,346,160 | 97.25 | | 51.77 | 91.29 |
| W51-U4 | 26,722,231 | 25,058,229 | 97.23 | | 51.86 | 91.71 |
