# Supplementary material for: Transcriptome profiling analysis of uterus during chicken laying periods
Source: BMC Genomics. 2023 Aug 3;24:433. doi: 10.1186/s12864-023-09521-z (PMC10398974; doi:10.1186/s12864-023-09521-z)
Supplement: Supplementary file 3 — Additional file 3. [file 12864_2023_9521_MOESM3_ESM.docx]

Supplementary table 3 Information of enriched GO Terms based on DEGs of W31 vs W22

| ONTOLOGY | GO Terms | DEGs No. | P-adjust | Genes |
| --- | --- | --- | --- | --- |
| CC | extracellular matrix | 15 | 0.000000 | COL1A2/COL1A1/WNT11/THBS2/FN1/TF/ELN/VCAN/TECTA/CCN3/COL3A1/NTN3/COCH/CYR61/WNT4 |
| CC | extracellular region part | 24 | 0.000015 | PTN/COL1A2/COL1A1/BMP4/WNT11/SOSTDC1/WNT9A/SPP1/MOXD1/THBS2/FN1/TF/ELN/VCAN/TECTA/CCN3/COL3A1/NTN3/COCH/LOX/CYR61/LECT2/WFDC1/WNT4 |
| CC | extracellular region | 27 | 0.000025 | PTN/KERA/AVD/COL1A2/COL1A1/BMP4/WNT11/SOSTDC1/WNT9A/SPP1/MOXD1/ACHE/THBS2/FN1/TF/ELN/VCAN/TECTA/CCN3/COL3A1/NTN3/COCH/LOX/CYR61/LECT2/WFDC1/WNT4 |
| CC | collagen-containing extracellular matrix | 8 | 0.000687 | COL1A2/COL1A1/THBS2/ELN/TECTA/COL3A1/NTN3/COCH |
| CC | extracellular space | 16 | 0.027378 | PTN/COL1A2/COL1A1/BMP4/WNT11/SOSTDC1/WNT9A/SPP1/MOXD1/TF/COL3A1/COCH/LOX/LECT2/WFDC1/WNT4 |
| MF | glycosaminoglycan binding | 7 | 0.004357 | PTN/SPP1/THBS2/FN1/VCAN/CCN3/CYR61 |
| MF | heparin binding | 6 | 0.004357 | PTN/SPP1/THBS2/FN1/CCN3/CYR61 |
| MF | sulfur compound binding | 7 | 0.004357 | PTN/AVD/SPP1/THBS2/FN1/CCN3/CYR61 |
| MF | extracellular matrix structural constituent | 5 | 0.007256 | COL1A2/COL1A1/ELN/TECTA/COL3A1 |

| ONTOLOGY | GO Terms | DEGs No. | P-adjust | Genes |
| --- | --- | --- | --- | --- |
| CC | extracellular region | 29 | 0.005033 | AvBD10/PTN/TNR/AVD/GREM1/FST/WNT11/SPP1/PI15/FGF1/GH/ST6GAL1/TNC/NPY/ACE/INHBB/CPZ/RSFR/TSKU/BMP2/SLC2A14/EXFABP/AVR2/RBP4/LYG2/COCH/AvBD12/VLDLR/CYR61 |
| CC | extracellular region part | 22 | 0.041434 | AvBD10/PTN/TNR/GREM1/FST/WNT11/SPP1/PI15/FGF1/GH/NPY/ACE/INHBB/CPZ/TSKU/BMP2/SLC2A14/EXFABP/RBP4/COCH/VLDLR/CYR61 |

Supplementary table 4 Information of enriched GO Terms based on DEGs of W51 vs W31

Supplementary table 5 Information of enriched KEGG pathway based on DEGs of W51 vs W31

| Pathways | DEGs No. | P-adjust | Genes |
| --- | --- | --- | --- |
| Neuroactive ligand-receptor interaction | 24 | 0.010766 | RXFP1/GRIA1/P2RX1/MCHR2/DRD4/UTS2B/GH/CHRM4/POMC/CHRNB3/GALR2/NPY/GABBR2/SS2/CHRNA4/HTR2B/GRIA3/P2RX2/PENK/GNRHR/LPAR1/HTR2A/VIPR1/LEPR |

Supplementary table 6 Information of enriched GO Terms based on DEGs of W51 vs W22

| ONTOLOGY | GO Terms | DEGs No. | P-adjust | Genes |
| --- | --- | --- | --- | --- |
| CC | collagen-containing extracellular matrix | 13 | 0.000002 | COL6A1/COL1A2/COL1A1/COL6A2/COL12A1/THBS2/COL6A3/LUM/ELN/COL3A1/SPARC/NTN3/RBP4 |
| CC | extracellular matrix | 16 | 0.000002 | COL6A1/COL1A2/COL1A1/COL6A2/COL12A1/THBS2/COL6A3/LUM/ELN/TSKU/VCAN/CCN3/COL3A1/SPARC/NTN3/RBP4 |
| CC | collagen trimer | 8 | 0.000076 | COL6A1/COL1A2/COL1A1/COL6A2/COL12A1/COL6A3/LUM/COL3A1 |
| CC | extracellular region | 33 | 0.000162 | COL6A1/CG-1B/AVD/COL1A2/COL1A1/GREM1/FST/COL6A2/FGF1/GH/PLAU/COL12A1/FGA/RNASE6/TNC/THBS2/INHBB/COL6A3/LUM/ELN/RSFR/TSKU/VCAN/CCDC80/CCN3/SEMA3D/COL3A1/SPARC/SLC2A14/NTN3/ZP3/RBP4/WFDC1 |
| CC | extracellular region part | 28 | 0.000162 | COL6A1/CG-1B/COL1A2/COL1A1/GREM1/FST/COL6A2/FGF1/GH/PLAU/COL12A1/FGA/THBS2/INHBB/COL6A3/LUM/ELN/TSKU/VCAN/CCN3/SEMA3D/COL3A1/SPARC/SLC2A14/NTN3/ZP3/RBP4/WFDC1 |
| CC | supramolecular complex | 16 | 0.008582 | ACTA1/F-KER/COL1A2/COL1A1/DES/TUBAL3/CSRP2/TUBB2B/MYOM2/PLS1/TNNT3/INCENP/LUM/ELN/COL3A1/ACTC1 |
| CC | supramolecular polymer | 16 | 0.008582 | ACTA1/F-KER/COL1A2/COL1A1/DES/TUBAL3/CSRP2/TUBB2B/MYOM2/PLS1/TNNT3/INCENP/LUM/ELN/COL3A1/ACTC1 |
| CC | supramolecular fiber | 16 | 0.008582 | ACTA1/F-KER/COL1A2/COL1A1/DES/TUBAL3/CSRP2/TUBB2B/MYOM2/PLS1/TNNT3/INCENP/LUM/ELN/COL3A1/ACTC1 |
| CC | extracellular space | 21 | 0.017900 | COL6A1/CG-1B/COL1A2/COL1A1/GREM1/FST/COL6A2/FGF1/GH/PLAU/COL12A1/FGA/INHBB/COL6A3/TSKU/SEMA3D/COL3A1/SPARC/SLC2A14/ZP3/WFDC1 |

Supplementary table 7 Information of enriched KEGG pathway based on DEGs of W51 vs W22

| Pathways | DEGs No. | P-adjust | Genes |
| --- | --- | --- | --- |
| ECM-receptor interaction | 15 | 0.000003 | COL6A1/COL1A2/COL1A1/DMP1/NPNT/COL6A2/ITGB6/TNC/THBS2/COL6A3/LAMB4/VTN/SDC1/FREM1/COL4A4 |
